# DamX Controls Reversible Cell Morphology Switching in Uropathogenic *Escherichia coli*

**DOI:** 10.1128/mBio.00642-16

**Published:** 2016-08-02

**Authors:** Surabhi Khandige, Cecilie Antoinette Asferg, Karina Juhl Rasmussen, Martin Jakob Larsen, Martin Overgaard, Thomas Emil Andersen, Jakob Møller-Jensen

**Affiliations:** a Department of Biochemistry and Molecular Biology, University of Southern Denmark, Odense, Denmark; b Department of Clinical Genetics, Odense University Hospital, Odense, Denmark; c Department of Human Genetics, Institute of Clinical Research, University of Southern Denmark, Odense, Denmark; d Department of Clinical Biochemistry and Pharmacology, Odense University Hospital, Odense, Denmark; e Research Unit of Clinical Microbiology, Odense University Hospital, Odense, Denmark

## Abstract

The ability to change cell morphology is an advantageous characteristic adopted by multiple pathogenic bacteria in order to evade host immune detection and assault during infection. Uropathogenic *Escherichia coli* (UPEC) exhibits such cellular dynamics and has been shown to transition through a series of distinct morphological phenotypes during a urinary tract infection. Here, we report the first systematic spatio-temporal gene expression analysis of the UPEC transition through these phenotypes by using a flow chamber-based *in vitro* infection model that simulates conditions in the bladder. This analysis revealed a novel association between the cell division gene *damX* and reversible UPEC filamentation. We demonstrate a lack of reversible bacterial filamentation in a *damX* deletion mutant *in vitro* and absence of a filamentous response by this mutant in a murine model of cystitis. While deletion of *damX* abrogated UPEC filamentation and secondary surface colonization in tissue culture and in mouse infections, transient overexpression of *damX* resulted in reversible UPEC filamentation. In this study, we identify a hitherto-unknown *damX-*mediated mechanism underlying UPEC morphotypical switching. Murine infection studies showed that DamX is essential for establishment of a robust urinary tract infection, thus emphasizing its role as a mediator of virulence. Our study demonstrates the value of an *in vitro* methodology, in which uroepithelium infection is closely simulated, when undertaking targeted investigations that are challenging to perform in animal infection models.

## IMPORTANCE

Urinary tract infections (UTIs) are most often caused by uropathogenic *Escherichia coli* (UPEC) and account for a considerable health care burden. UPEC exhibits a dynamic lifestyle in the course of infection, in which the bacterium transiently adopts alternative morphologies ranging from rod shaped to coccoid and filamentous, rendering it better at immune evasion and host epithelium adhesion. This penchant for morphotype switching might in large measure account for UPEC’s success as a pathogen. In aiming to uncover genes underlying the phenomenon of UPEC morphotype switching, this study identifies *damX*, a cell division gene, as a mediator of reversible filamentation during UTI. DamX-mediated filamentation represents an additional pathway for bacterial cell shape control, an alternative to SulA-mediated FtsZ sequestration during *E. coli* uropathogenesis, and hence represents a potential target for combating UTI.

## INTRODUCTION

The success of a bacterial pathogen depends on its ability to sense its environment and adapt suitably to ensure the best odds of survival. One such adaptation that bestows a selective advantage on the pathogen is its potential for morphological plasticity (1–3). Bacteria have been known to adopt a filamentous morphology when subjected to stresses such as starvation (4), pH change (5), low water activity (6), and exposure to antimicrobial agents (7).

Uropathogenic *Escherichia coli* (UPEC) has evolved a characteristic lifestyle within the host that enables efficient immune evasion and renders it a successful pathogen. UPEC is the most common cause of urinary tract infections (UTIs) (8), and these bacteria have been observed to undergo morphological differentiation during infection in mice (9) and humans (10). In the course of an acute UTI, the normally rod-shaped UPEC adheres to and invades the superficial cells of the bladder urothelium. Invading UPEC replicates within intracellular bacterial communities (IBCs), consisting mainly of coccoid bacteria (11). Eventually, the intracellular bacterial burden leads to host cell death, at which point UPEC bacteria leave the host cell in the form of motile rods as well as highly filamentous bacteria. The UPEC filaments released into the extracellular milieu possess the ability to revert to the original rod-shaped morphology with the potential to reinitiate a new cycle of infection. Although knowledge of the UPEC infection cycle is derived mainly from murine models of cystitis, IBCs and UPEC filaments have been detected in urine samples from humans with acute UTI (10). The ability to revert to normal rod-shaped morphology is as critical as the ability to form filaments, not just in the case of bacteria like UPEC that adopt filamentation as a virulence strategy but also in cases where filamentation is a result of sublethal stress (1). Studies have shown that filamentation of pathogenic bacteria is likely either part of a strategy that helps the pathogen evade the host innate immune response (9, 12) or a stress coping mechanism within a hostile host (13).

The phenomenon of bacterial filamentation is, in essence, a consequence of stalled cell division in the bacterial cell. Cell division, a complex process involving ~30 essential and nonessential genes (14), comprises a sequence of events that commences with the assembly of a membrane-tethered cytoplasmic proto-ring (15). The proto-ring comprises FtsZ, FtsA, and ZipA, which represent the earliest recruits to the septal plane (16), followed by other proteins that build on the proto-ring after a considerable time lag (17). The final step in cell division involves cell wall peptidoglycan remodeling, i.e., degradation of preexisting peptidoglycan and synthesis of septal peptidoglycan (18), which ultimately results in cell wall invagination and the formation of two daughter cells.

While some studies have aimed at identifying genes involved in bacterial filamentation (13, 19–21), this study is the first to systematically analyze the UPEC gene expression profiles at each individual phase of its pathogenesis, with the aim of identifying genes that are critical to its morphological differentiation. Using microarray technology and a previously described (22) flow chamber-based bladder cell infection model, this study not only reaffirms recent reports regarding the importance of a contact surface and fluid flow in UPEC filamentation (23) but also identifies DamX as a key factor that controls reversible switching between normal rod-shaped and filamentous morphology during infection. While previous studies have linked UPEC filamentation with the SOS response and SulA (9, 12, 20), our present study identifies transient DamX overproduction as a key inducer of filamentation during this cycle. Based on the currently available data, we propose that both mechanisms act in concert to create the extensive filamentation observed *in vivo* but that they are triggered by different mechanisms: the DamX filamentation triggered as a response to the shift from intracellular growth to urine- and liquid shear-exposed surface growth and the SOS filamentation triggered by immune cell attack (12).

## RESULTS

### Expression analysis revealed differential UPEC transcription profiles during *in vitro* infection.

**FIG 1:**
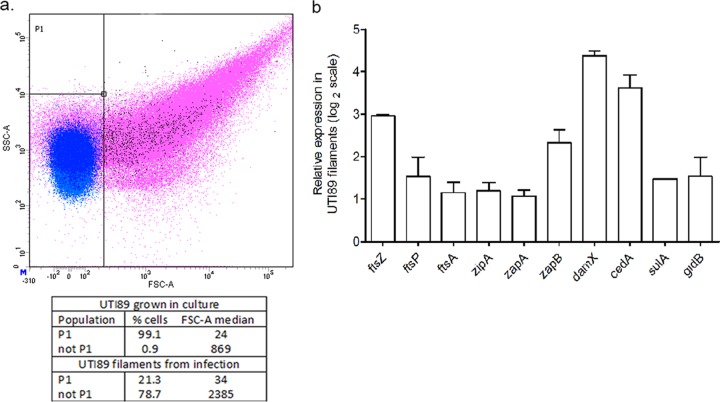
Expression of cell division genes in UTI89 filaments. (a) The fraction of filamentous bacteria in the population was ascertained by flow cytometry analysis. The scatterplot was gated based on the population of normal rod-shaped bacteria, with gate P1 representing normal rod-shaped bacteria from strain UTI89 (blue) and gate “notP1” representing UTI89 filaments (pink), with forward scatter and side scatter on the *x* and *y* axes, respectively. (b) The expression levels of cell division genes that were found to be differentially expressed between filaments and the urine reference condition were validated by real-time PCR. The graph represents mean expression values of UTI89 in the filament phase relative to expression levels under the reference condition of strain UTI89 cultured in urine, expressed as the fold change on a log_2_ scale.

The infection cycle of the UPEC strain UTI89, originally isolated from a case of cystitis (24), has been well characterized since early reports more than a decade ago of UPEC intracellular behavior during infection (25, 26). Characterization of the genes responsible for progression of the UPEC infection cycle has been limited by the obvious challenges of low bacterial yields from *in vivo* infection models and the inability to recapitulate the cycle *in vitro.* We recently established an *in vitro* infection model capable of supporting the morphological changes inherent to progression of the UPEC infection cycle (22). Working on the assumption that the morphological transitions observed in our *in vitro* model reflect many of the characteristics of an *in vivo* infection, we explored the unique opportunity offered by this model to harvest and analyze bacteria from distinct phases of infection. Systematic transcriptional profiling of UTI89 obtained from the intracellular, filamentous, and filament reversal (here referred to as reversal) phases was performed using microarray technology (illustrated in Fig. S1a in the supplemental material). A custom-designed UTI89-specific array was used to identify both annotated and unannotated transcripts covering the entire UTI89 genome (accession number NC_007946.1) as well as that of pUTI89 (accession number NC_007941.1). Bacterial cells harvested from the flow chamber apparatus representing the filamentous and reversal phases were isolated from three independent flow chamber experiments and used for hybridization as biological triplicates on the UTI89 array. In order to ascertain the fraction of the sample that was truly filamentous, UTI89 was harvested after growth under two conditions—a reference condition that resulted in rod-shaped UTI89 cells grown in LB culture and a filamentous condition from within the flow chambers during the filamentous phase of infection—and analyzed using flow cytometry. Gating was performed on the culture-grown, normal rod-shaped bacteria, resulting in the populations “P1” and “not P1,” such that rod-shaped bacteria were represented in the former and filaments were represented in the latter populations. Examination of the two populations under the two conditions revealed that ~80% of the flow chamber sample was filamentous, with an FSC-A median (forward scatter area, a measure of cell length) of 2,385, compared to an FSC-A median of ~30 in the nonfilamentous rod-shaped P1 population (Fig. 1a). Since the filamentous phase also contains a small fraction of rod-shaped bacteria, it is possible that expression levels in this phase are underestimated.

Due to the relatively low yield of bacterial mRNA from the intracellular phase, samples were pooled from four parallel flow chambers in three independent experiments to yield biological triplicates. In order to obtain reference conditions that were unbiased toward a particular growth environment, bacteria were cultured to stationary phase in nutrient-rich Luria-Bertani (LB) broth representing the growth medium at the time of infection, as well as sterile human urine with a urine specific gravity (USG; the density) of ≥1.025, representing the flow medium during *in vitro* infection. The choices of growth phase and culture medium for the reference condition were admittedly challenging, since the UPEC growth phase and nutrient sources are likely subject to frequent changes during intra- and extracellular growth in the flow chamber model.

Gene transcripts from intracellular, filamentous, and reversal phases were filtered on the basis of the *P* value and log_2_ fold change in expression, individually, compared to the reference conditions, urine and LB broth. Among the transcripts that crossed the fold change and *P* value thresholds, those that demonstrated disparate expression trends (up- or downregulated) compared to LB and urine cultures were excluded from further analysis. These transcripts would more likely represent a response to the particular growth environment (LB/urine). Only gene transcripts from each of the three phases that showed consistent trends of differential expression, irrespective of the reference used (LB/urine), were considered further.

A total of 351 transcripts in intracellular phase, 845 in filamentous phase, and 447 in reversal phase were found to display a statistically significant difference in *t* test results, i.e., *P* values of ≤0.05 and fold changes of ≥2 log_2_ compared to the urine cultured reference condition. Among the significant differentially expressed transcripts, 27% of those in the intracellular phase, 33% in the reversal phase, and 75% in the filamentous phase were found to be uniquely differentially expressed during each particular phase of infection. Examination of the significant transcripts revealed that the expression profiles during the intracellular and reversal phase were more comparable, while the expression profile during the filamentous phase showed not only a deviation from the other two phases but also a state of increased transcriptional activity (reflected in the higher number of upregulated genes that passed the statistical threshold). Gene ontology distribution of the significant transcripts showed an enrichment of genes involved in metabolic processes and cell division in the filamentous phase of infection (see Fig. S1c in the supplemental material).

### Deletion of *damX* resulted in abrogation of the filamentation phenotype *in vitro.*

The observation that cell division was among the ontologies differentially expressed in the filamentous phase of UTI89 (see Fig. S1c in the supplemental material) was not unexpected, since bacterial filamentation essentially results from an absence of cytokinesis. The array data revealed a cluster of genes upregulated in filaments that included several cell division genes involved in septal ring formation, like *zapB* (5.2-fold log_2_ change), *ftsZ* (6.6-fold log_2_), *ftsA* (3.2-fold log_2_), and *cedA* (8.4-fold log_2_), all of which were correspondingly 2- to 6-fold log_2_ downregulated in the preceding intracellular and subsequent reversal phases (see Fig. S1b). The observation that genes encoding proto-ring components *ftsZ*, *ftsA*, and *zipA* that were upregulated in filaments indicated that a halt in cell division probably occurred downstream of proto-ring assembly. In addition to some essential and nonessential division genes, two genes belonging to the SPOR domain class of proteins (*damX* and *rlpA*) were found to be significantly upregulated in filaments (6-fold log_2_ and 4.2-fold log_2_, respectively). Conversely, genes encoding other known SPOR domain proteins, FtsN and DedD, were not significantly upregulated during filamentation. Real-time PCR was performed to validate expression levels of several of the cell division genes; data are presented as expression levels in UTI89 filaments relative to expression in a stationary UTI89 urine culture (Fig. 1b).

**FIG 2:**
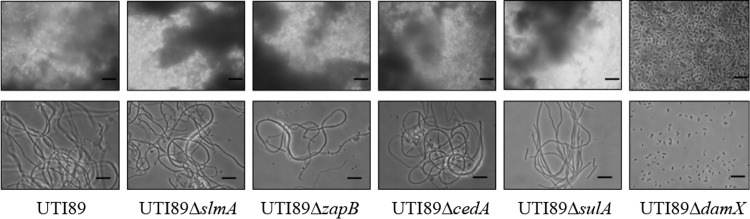
Strain UTI89Δ*damX* was unable to demonstrate secondary infection or filamentation on urine exposure. Flow chamber-based infection of the PD07i bladder cell line was carried out with strains UTI89wt, UTI89Δ*slmA*, UTI89Δ*zapB*, UTI89Δ*cedA*, UTI89Δ*sulA*, and UTI89Δ*damX*. Panels in the top row represent secondary infection and biofilm formation (cloudy appearance) on exposure to concentrated urine, and the bottom row depicts bacteria flushed out of the flow chambers after 22 h of concentrated urine exposure. Scale bars, 20 µm.

Based on our findings and previous studies linking cell division genes to a filamentous morphology, *sulA* (9, 27), *slmA* (28), *zapB* (29), *cedA* (30), and *damX* (31) were chosen for further exploration in the context of UPEC filamentation. Individual deletion strains for the genes were constructed in UTI89 in order to evaluate their influence on filamentation in the flow chamber infection model. Strains UTI89Δ*cedA*, UTI89Δ*zapB*, UTI89Δ*sulA*, UTI89Δ*damX*, and UTI89Δ*slmA* were all found to demonstrate growth rates comparable to UTI89 wild type when cultured in LB broth and urine (see Fig. S2a in the supplemental material), confirming viability of the respective deletion strains. All five deletion strains were subsequently tested along with the UTI89 wild type for their ability to form filaments and secondary surface colonization, i.e., phenotypes originating from an intracellular reservoir post-gentamicin treatment, in the flow chamber infection model (Fig. 2a). We observed that while UTI89Δ*cedA*, UTI89Δ*zapB*, UTI89Δ*sulA*, and UTI89Δ*slmA* mutant strains behaved similarly to wild-type UTI89, demonstrating both secondary surface colonization and concurrent filament formation, the UTI89Δ*damX* strain was unable to produce both phenotypes.

In order to investigate whether the lack of filamentation was merely due to an inability of strain UTI89Δ*damX* to adhere to and invade the bladder epithelial cell line, UTI89Δ*damX* mutant cells were tested in a stationary infection setup performed using the bladder epithelial cell line PD07i in multiwell plates. These results showed that the UTI89Δ*damX* mutant efficiency equaled that of the wild type in early bladder cell adhesion and invasion in short-term *in vitro* infection (see Fig. S2b and c in the supplemental material). Additionally, to assess possible intracellular growth defects in the UTI89Δ*damX* mutant, we performed a plasmid retention assay using a temperature-sensitive low-copy-number plasmid, pKD46 (32). The percentage of plasmid-retaining bacterial cells at 24 h postinfection (hpi) was found to be on average around 70% in strain UTI89/pKD46 and 72% in strain UTI89Δ*damX*/pKD46, demonstrating that both strains underwent similar numbers of intracellular division events (see Fig. S2d) and indicating that *damX* deletion does not lead to intracellular growth attenuation. We further plotted a standard curve of the percent retention of the pKD46 plasmid along the growth curve, as a function of time, to estimate the average number of intracellular generations. Both the UTI89 and UTI89Δ*damX* strains grew to an average of 4 to 5 generations intracellularly (based on the doubling times depicted in Fig. S2a), as depicted in Fig. S2e.

Recent studies have reported that *damX*, first identified as *urf74.3* by Lyngstadaas et al. (31), encodes a SPOR domain-containing protein that is recruited to the septum during cell division (33, 34). SPOR domains are found in many bacterial proteins and are involved in binding to septal peptidoglycan (35–37). Overproduction of DamX has been reported to result in a failure in cell division and a filamentous morphology, while deletion of the *damX* gene has been associated with bile sensitivity (38) without a discernible impact on cell morphology (34). *damX* is part of a functionally diverse operon comprised of *aroBK*, *damX*, *dam*, *rpe*, *gph*, and *trpS*. Some previous studies suggested that polarity effects of *damX* deletion on the downstream *dam* gene, which encodes the Dam DNA methylase, could account for the phenotypes associated with *damX* deletion, due to a likely σ^70^ *damp1* promoter within the *damX* open reading frame (39–41). Analysis of *dam*, *aroBK*, and *damX* expression levels in the UTI89Δ*damX* mutant relative to the UTI89 wild type revealed no significant effect of *damX* deletion on *dam* expression (see Fig. S3a in the supplemental material)*.* In order to confirm with certainty the absence of any polarity issues, we constructed and tested a more subtle deletion mutant, UTI89ΔdamX*, in which the *damp1* promoter was left intact. The UTI89ΔdamX* deletion strain replicated the filamentation defect displayed by strain UTI89Δ*damX* during the flow chamber-based infection experiments (see Fig. S3b). This finding indicated that the lack of filamentation observed in strain UTI89Δ*damX* was more likely attributable directly to the deletion of *damX* rather than secondary *dam*-mediated effects.

### The UTI89Δ*damX* mutant is unable to form filaments in an *in vivo* mouse model.

In order to validate our findings from the *in vitro* flow chamber model and assess the role of DamX as a mediator of UPEC filamentation within a complex *in vivo* environment, a mouse cystitis model based on the strain C3H/HeN was chosen for further investigation. C3H/HeN mice are known to be fully immunocompetent, with a wild-type *tlr-4* gene and a functional PMN (polymorphonuclear leukocyte) response. This mouse strain has been reported to support a robust UPEC pathogenesis cycle in several previous studies (25).

**FIG 3:**
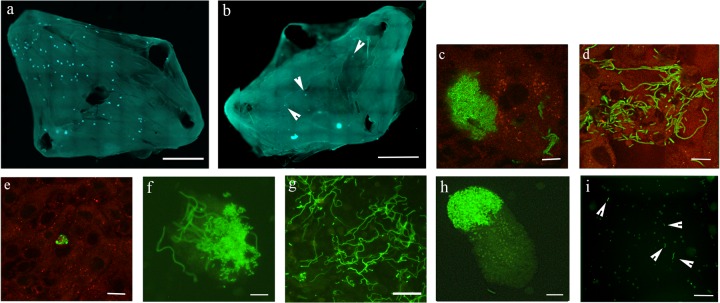
Strain UTI89Δ*damX in vivo* infection reflects the failure to form filaments *in vitro*. Splayed urinary bladders of C3H/HeN mice were examined for IBCs at 12 hpi in strains UTI89/pMAN01 (a) and UTI89Δ*damX*/pMAN01 (b) and for filaments also at 18 hpi for strains UTI89/pMAN01 (c and d) and UTI89Δ*damX*/pMAN01 (e). White arrowheads in panel b denote IBCs. Urine collected from strain UTI89/pMAN01-infected bladders at 12 hpi, prior to organ harvest, was examined and revealed sloughed epithelial cells with IBCs (f) as well as bacterial filaments (g). Urine examined after strain UTI89Δ*damX*/pMAN01 infection, 12 hpi, also revealed sloughed epithelial cells with IBCs (h) but only rod-shaped bacteria (i). White arrowheads in panel “i” point to individual bacteria. Scale bars, 1 mm (a and b) and 10 µm (c to i).

Female C3H/HeN mice were separately inoculated via transurethral catheterization with strain UTI89/pMAN01 or UTI89Δ*damX*/pMAN01 subcultured over two rounds of static growth at 37°C in LB medium. The strains were transformed with the pMAN01 plasmid to ensure constitutive expression of *gfp*, enabling easy microscopic examination of the bacteria. The mouse bladders at 12 and 18 hpi as well as urine collected immediately prior to tissue harvest at 12 h were subjected to microscopic examination for bacterial filaments, exfoliated cells, and IBCs. Alongside this, CFU counts were estimated in bladder homogenates at 18 h, 48 h, and 14 days postinfection. At the 12-h time point, several IBCs were observed in bladders infected with strain UTI89Δ*damX*, although they appeared to be fewer in number than in wild-type-infected bladders (Fig. 3a and b). Inspections of urine specimens at this time point revealed relatively high numbers of exfoliated cells containing IBCs also from the mutant strain infections (Fig. 3h), showing that IBCs are formed by strain UTI89Δ*damX*, but they are cleared more rapidly than the wild-type strain.

At 18 hpi, IBCs were largely cleared from bladders infected with strain UTI89Δ*damX*, whereas IBCs were still present in wild-type-infected bladders (Fig. 3c and e). The wild-type strain, UTI89wt, exhibited abundant filamentation (Fig. 3d), whereas no filaments were detected in the bladders infected with strain UTI89Δ*damX*. Mutant UTI89Δ*damX* cells were, however, few and far apart on the bladder lumen wall, making it difficult, based on these inspections alone, to conclude on a possible filamentation defect. By pooling urine samples from strain UTI89Δ*damX*-infected mice, much higher numbers (thousands) of bacteria could be analyzed. Strain UTI89Δ*damX* in these samples were all found to be normal rod shaped or only slightly elongated (Fig. 3i), which was in stark contrast to the highly filamentous response present in the urine from UTI89wt strain infections (Fig. 3g).

**FIG 4:**
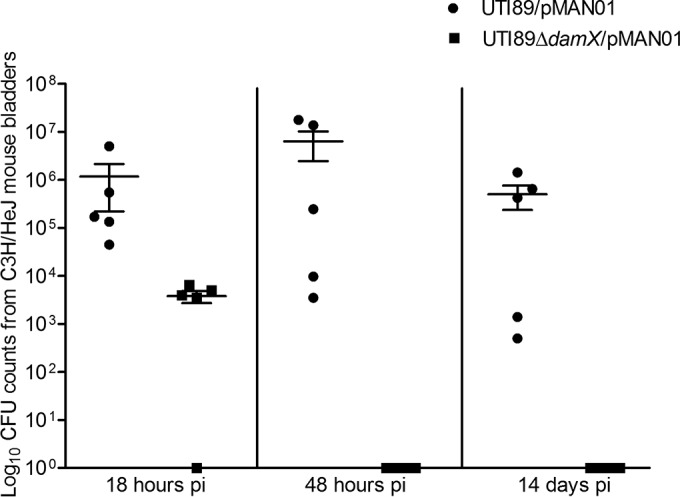
Enhanced clearance of strain UTI89Δ*damX* infection from C3H/HeN bladders. Bladders harvested from C3H/HeN mice infected with strain UTI89/pMAN01 (circles) or strain UTI89Δ*damX*/pMAN01 (squares) 18 hpi, 48 hpi, and 14 days postinfeciton were homogenized, and suitable dilutions were plated for CFU counts. The 10^0^ data on the *y* axis of the graph are equivalent to a finding of no detectable bacteria during CFU counting. Infections were carried out in 5 mice per group in two independent rounds of experimentation. A Mann-Whitney test was applied to calculate statistical significance, and the *P* value was found to be <0.05.

Regarding overall bacterial numbers in the bladders, CFU counts were approximately 2.5 orders of magnitude lower for strain UTI89Δ*damX*/pMAN01 than for the UTI89/pMAN01 strain at 18 hpi (Fig. 4), thus indicating a significant defect in progression of infection beyond the initial intracellular infection cycle.

CFU counts measured at 48 h and 14 days postinfection revealed a severely diminished capacity for persistence of strain UTI89Δ*damX*/pMAN01 during bladder infection. The CFU detection limit was set at 50 bacteria per bladder, implying that a count of <50 bacteria was equivalent to complete clearance of infection. A deletion of *damX* resulted in clearance of the infection at 48 hpi, whereas the UTI89/pMAN01 strain presented high CFU counts in the mouse bladders even at 14 days postinfection (Fig. 4).

Previously, Justice et al. (9) proposed a *sulA*-driven SOS-mediated response as a mechanism underlying UPEC filamentation during the uropathogenic cycle. While our *in vitro* infections failed to show a correlation between *sulA* and UPEC filamentation (Fig. 2), this could have been due to the absence of immune cell attack in this model, which has been hypothesized to underlie the SOS-triggered filamentation (9). To be able to better investigate an *in vivo sulA-*filamentation association that perhaps could not be observed in the *in vitro* flow chamber model, we performed infections of the C3H/HeN mouse strain with strain UTI89Δ*sulA*/pMAN01. A visual examination of splayed bladders 18 hpi revealed a scenario akin to UTI89Δ*damX* strain infection, with very few or no IBCs and no detectable filaments (see Fig. S4a in the supplemental material). Notwithstanding the overall poor bacterial load in these urine samples, our examination of voided urine collected prior to bladder harvest revealed several long filaments along with short rods (see Fig. S4b and c).

### Variation in *damX* expression levels resulted in reversible filamentation.

**FIG 5:**
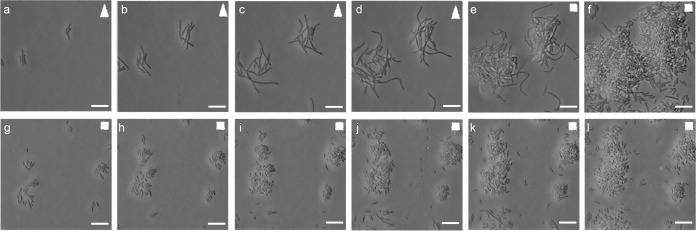
Overexpression of *damX* induces reversible UTI89 filamentation. Time-lapse images of strain UTI89/pSKdamX seeded on glass slides subjected to a flow of medium supplemented with 0.2% arabinose (white triangles) shown (a to d) or 0.4% glucose (white squares) (e and f), sequentially. (g to l) A negative control in which strain UTI89/pSKdamX was only subjected to 0.4% glucose (white squares) mediated *damX* repression. Addition of 0.2% arabinose (white triangles) allowed *damX* overexpression and filamentation therein, as seen in panels a to d. This filamentation phenotype was lost when *damX* induction was turned off, as seen in panels e, f, and g to l. Images were collected at 20-min intervals. Scale bars, 20 µm.

In order to prove that transient DamX overproduction is key to reversible UTI89 filamentation, we cloned the UTI89 *damX* gene with a strong upstream Shine-Dalgarno sequence in pBAD33, to form pSKdamX, thereby allowing for arabinose-induced expression and subsequent tight repression by glucose addition. Previously, Lyngstadaas et al. (31) reported that overproduction of DamX was detrimental to cell growth. Initial attempts to induce *damX* overexpression during growth in liquid medium proved more challenging than anticipated and did not yield filaments. Unlike the earlier flow chamber experiments that simulated bladder infection, examination of the effect of *damX* overexpression from pSKdamX was performed by seeding UPEC on an abiotic glass surface, for ease of handling. Strain UTI89/pSKdamX was therefore cultured overnight on LB agar plates supplemented with 0.4% glucose to ensure complete repression of *damX* expression from pSKdamX and seeded onto sterile glass slides inserted into the flow chamber. After 20 min of seeding, the chambers were exposed to a flow of EZ medium supplemented with 0.2% arabinose. Microscopy images captured at 10-min intervals clearly demonstrated the effect of *damX* overexpression on UTI89 cell morphology (Fig. 5a to d). Since the morphological plasticity observed during infection entails not only the ability to form filaments but also the ability for those filaments to revert to normal rod-shaped bacteria (25), it was important to demonstrate that termination of *damX* overexpression with the subsequent addition of 0.4% glucose would facilitate UTI89 filament reversal. This was indeed our observation (Fig. 5e and f). Strain UTI89/pSKdamX exposed exclusively to EZ plus 0.4% glucose was observed in parallel in order to clearly demonstrate that UTI89 filamentation was indeed a result of *damX* overexpression. As seen in Fig. 5g to l, this control experiment yielded a complete absence of filaments. Concurrently, the vector control strain UTI89/pBAD33 was also tested in the presence of 0.2% arabinose and was found not to form filaments (see Videos S1a to c in the supplemental material).

**FIG 6:**
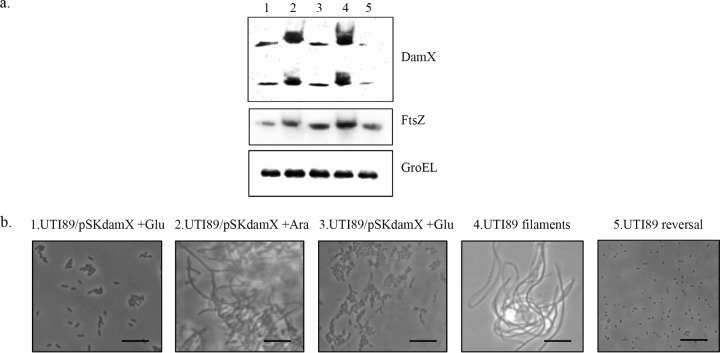
The UTI89 morphological state is reflected in DamX protein levels. (a) Western blot analysis of DamX was performed. Lanes: 1, strain UTI89/pSKdamX under 0.4% glucose repression prior to seeding on glass slides; 2, strain UTI89/pSKdamX filaments induced by 0.2% arabinose; 3, strain UTI89/pSKdamX filament reversal under 0.4% glucose; 4, native UTI89 filaments; 5, reversed native filaments. Both of the upper bands correspond to DamX (34), the middle panel corresponds to FtsZ levels, and the bottom panel shows GroEL expression, which was used as the internal loading control. (b) Microscopic examination of UTI89 strains from each of the respective samples from lanes 1 to 5 was performed to confirm morphological status. Scale bars, 20 µm.

In light of preexisting knowledge that *damX* overexpression results in *E. coli* K-12 filamentation and that the pBAD system is admittedly an artificial setup, a comparison of DamX levels between native UTI89 filaments flushed out of the flow chamber model and artificial arabinose-induced UTI89/pSKdamX strain filaments would be critical to validation of this experiment. By Western blot analysis, the DamX level in arabinose-induced UTI89/pSKdamX filaments was found to be elevated and comparable to that of native UTI89 filaments obtained from a flow chamber infection experiment (Fig. 6a). This was in contrast to uninduced UTI89/pSKdamX and rod-shaped UTI89 cells resulting from filament reversal, which contained much lower DamX levels.

Both array and real-time PCR data revealed elevated levels of *ftsZ* expression in native UTI89 filaments*.* Based on prevailing knowledge linking *ftsZ* overexpression to a filamentous phenotype, we then proceeded to determine FtsZ levels in these samples by Western analysis. We found that FtsZ levels were elevated in the native filaments (Fig. 6a, lane 4) compared to reverted filaments (lane 5), as expected from our transcriptional data. Concurrently, while we observed an increase in FtsZ levels in the arabinose-induced filaments (lane 2) compared to the preinduction rods (lane 1), there was no discernible drop in FtsZ levels in glucose-induced reverted filaments (lane 3). This suggested that UTI89 filaments were not merely due to *ftsZ* overexpression and, second, that *damX* overexpression was not directly linked to *ftsZ* overexpression. Microscopic examination was performed to confirm the morphology of bacteria flushed out of the flow chambers from each of the five samples used for Western blotting (Fig. 6b).

## DISCUSSION

While the survival advantage conferred by morphological differentiation on UPEC infection has been recognized (9, 12), the precise mechanisms underlying this phenomenon have not been resolved thus far. This study adopted a novel approach that allowed for transcriptional profiling of distinct phases of UPEC infection and thereby identified a relatively unexplored cell division protein, DamX, as critical to reversible UPEC filamentation.

The understanding of UPEC pathogenesis has grown considerably since the earliest reports detailing the various stages of infection observed in murine cystitis models (11, 25). UPEC has been known to demonstrate a complex infection cycle in the bladder, commencing with adhesion and invasion of the superficial bladder epithelial cells (24), within which the bacteria replicate to form dense intracellular communities of coccoid bacteria, classified as IBCs. The model proposes that a subpopulation within these IBCs then undergoes a process of maturation to form filaments that undergo flux from the infected epithelial cell, and subsequently reverts to normal rod-shaped bacteria that hold the potential to reinitiate a fresh cycle of infection. This cyclic transition between intra- and extracellular localization along with a concurrent transition between rod-shaped, coccoid, and filamentous cell morphologies provides a mechanistic framework for explaining the high degree of recurrences observed among infected individuals (42). Observations from this and previous studies (9) suggest that the filamentous phase of UPEC infection must be transient and reversible in order to ensure progression of UTIs from the primary episode. In this study, we observed that strain UTI89Δ*damX* was incapable of forming filaments either *in vitro* or *in vivo.* The mutant strain displayed no reduction in bladder cell adhesion or invasion *in vitro*. Furthermore, the rate of strain UTI89Δ*damX* replication was comparable to that of the wild type both when cultured in human urine and when residing intracellularly *in vivo*. Indeed, we detected exfoliated cells containing late-stage IBCs in mouse urine after infection by strain UTI89Δ*damX* (Fig. 3h)*.* Collectively, our *in vitro* and *in vivo* infection data point toward a function for DamX in the late stages of a primary infection cycle, i.e., when filament formation may be required for maintenance and spread of the initial colonization.

While in the extracellular space, facing the combined threats of elimination through bladder voiding (micturition) or phagocytosis by polymorphonuclear leukocytes recruited to the bladder lumen surface at the point of infection, the adoption of a filamentous morphology can be advantageous both in terms of tissue adherence and immune evasion. Such morphological plasticity has been known to offer selective advantages, such as host immune evasion by UPEC (9, 12) and *Proteus mirabilis* (43), antibiotic resistance in *Burkholderia pseudomallei* (44), and stress resistance in *Caulobacter crescentus* (45). By employing an *in vitro* flow chamber infection model (22) with the ability to recapitulate UPEC behavior reported during *in vivo* murine infection, UPEC could be harvested from the three main phases of infection—intracellular, filamentous, and reversal. Hence, this study further emphasizes the utility of the flow chamber infection model as a tool allowing for systematic analysis of bacterial gene expression in the course of infection.

Bacterial cells elongate to form filaments when there is a failure to complete cell division after segregation of nucleoids has occurred. Our comparison of UTI89 transcriptional profiles during the three infection phases revealed temporal expression of several genes involved in cell division, indicating a change in the expression levels of cell division genes during filamentation compared to intracellular and reversal phases, during which cell division would be expected to occur more or less normally. Previous fluorescence microscopic inspection of UPEC filaments generated in flow chambers revealed segregated nucleoids and partial septa (22), suggesting that the arrest in cell division occurred at a stage subsequent to proto-ring formation by FtsZ, FtsA, and ZipA. We hypothesize that the elevated expression of transcripts of other divisome proteins (like ZapA, ZapB, ZapC, FtsX, FtsK, and FtsL) observed in the array data, as well as the enrichment of the cell division gene ontology (see Fig. S1b and S2 in the supplemental material) could reflect filaments poised for initiation of cell division as soon as the relevant environmental cue is perceived.

Justice et al. (9) previously suggested that SulA could underlie the process of UPEC filamentation in an immunocompetent host with the ability to mount a host immune response and thereby incite a bacterial SOS response. Such evidence for SulA-mediated filamentation has also been reported in other pathogens, like *Listeria monocytogenes* (46) and *Salmonella enterica* serovar Typhimurium (47), under the onslaught of phagocytic immune cells. In agreement with previous flow chamber model experiments (22), we did not observe filamentation defects in strain UTI89Δ*sulA*, indicating that this gene is not responsible for the filamentation observed during secondary surface colonization *in vitro.* Among the candidate mutant strains tested using the flow chamber model in this study, strain UTI89Δ*damX* was the only one to display an absence of filamentation as well as secondary surface colonization of the bladder epithelial cells. However, these results do not rule out that recruitment and interaction with neutrophils contributes to the induction of UPEC filamentation *in vivo*. UPEC may therefore adopt more than one strategy to attain a filamentous morphology, triggered by an SOS stress response during an immune attack as well as a parallel DamX-mediated induction triggered by the general physicochemical and hydrodynamic growth conditions present in the bladder.

DamX is recruited to the midcell in an FtsZ-dependent manner (33, 34) and interacts directly with septal peptidoglycan via its C-terminal SPOR domain (36, 37). While deletion of *damX* was found not to affect cell division in this or previous studies (33, 34), overproduction of DamX has been linked to a filamentous phenotype in the past (31, 48). By using a bacterial two-hybrid screen, DamX has been shown to interact strongly with itself, FtsQ, and another SPOR domain-containing cell division protein, FtsN (33). Z ring maturation and cell division have been described as linear, dependent pathways, where perturbations in early proteins influence assembly of later “downstream” proteins (49), and in this context FtsN is believed to be the final recruit that links Z ring maturation and septal peptidoglycan synthesis, as detailed in a recent review by Weiss (50). Based on reported evidence that FtsN and DamX both localize to the septal peptidoglycan by means of their SPOR domains (34), it is tempting to speculate that DamX at elevated levels could competitively inhibit FtsN self-accumulation and block molecular interactions between essential cell division proteins and/or septal peptidoglycan synthesis. However, findings by Busiek and Margolin (51) demonstrated that FtsN was capable of interacting with FtsA (an essential proto-ring component) in a SPOR-independent manner, and since the FtsN and DamX SPOR domains themselves show <20% identity conservation (35), the mechanism of DamX-mediated filamentation might be more complex than a mere case of competition. An alternative hypothesis is that DamX sequesters FtsQ (33), a Z ring protein (15) known to increase septal peptidoglycan turnover via FtsN-FtsA interactions (52). The precise localization of Z ring components during filament formation and reversal and the role that DamX overproduction plays in this scheme remain subjects for future study that we are currently pursuing.

The *dam* operon is comprised of seven functionally diverse members, one of which is *damX*. Despite the positioning of alternative *dam* promoters within the *damX* coding region (40), deletion of *damX* did not have any significant bearing on Dam methylase expression, thereby excluding the possibility that the lack of filamentation observed for strain UTI89Δ*damX* in flow chamber infections was due to a polarity effect on Dam*.* Studies that have associated deletion of *damX* with a reduced invasion capacity in *S. enterica* (53) and induction of the SOS response in *E. coli* (54) were conducted on *damX* mutants generated using transposon insertion and therefore likely to have suffered *dam* polarity effects. The facts that strain UTI89Δ*damX* invaded bladder epithelial cells as efficiently as the wild type but was defective with respect to both secondary surface colonization and filament formation upon exposure to concentrated urine in the flow chamber model suggest a role for DamX in UPEC secondary infection. The exact mechanistic role of DamX-mediated filamentation needs further clarification; however, the defect in secondary surface colonization corresponds with a role of filamentation in urothelial cell adherence under hydrodynamic conditions, as suggested previously (9, 22).

Examination of filaments that were artificially induced from the pSKdamX construct and those native filaments obtained during the course of a flow chamber infection revealed comparable DamX protein levels. DamX levels in filaments (both induced and native) that had reverted to rod shape were also consistent among themselves and significantly lower than their filamentous counterparts. The effective corroboration of DamX levels with the corresponding morphological state in each of the UTI89 samples tested further strengthens the postulation of DamX as a mediator of UPEC morphological plasticity. Induction of UPEC filamentation via transient DamX overproduction from plasmid pSKdamX occurred only when seeded on a solid surface, thus implying a role for surface sensing and possibly fluid flow, irrespective of whether that surface is biotic (bladder epithelial cells) or abiotic (glass). This dependency of UPEC filamentation on surface growth has been confirmed in other recent studies of UTI89 grown in flow chambers (22, 23), implying that the process of filamentation does not restrict itself exclusively to the UPEC infection cascade.

We compared the phenotypes resulting from *damX* overexpression in K-12, for which earlier studies had shown filament formation in liquid culture, to UPEC, for which liquid culture failed to result in filament formation even in concentrated urine. Despite the high degree of sequence conservation between K-12 and UTI89, the prerequisite for a solid surface for filamentation clearly applied only to UTI89 (see Fig. S5 in the supplemental material). The reason for this difference is unknown, but it is tempting to speculate that UPEC-specific surface fimbriae might be implicated in controlling *damX* induction. Leclerc et al. (53) reported the failure of osmolarity, pH, and oxygen tension as triggers of *damX* overexpression in their study of *S. enterica*. Taken together with the surface dependence of stable DamX-mediated filaments observed in our study, upstream activators of *damX* expression and hence UPEC filamentation could be those involved in surface sensing. However, such a mechanism might seem at odds with the absence of UPEC filaments in bladders derived from infected immunocompromised mice reported by Justice et al. (9). Therefore, we cannot exclude a role of the host immune system in the induction of DamX-mediated filaments.

The Cpx two-component system monitors biogenesis of bacterial surface structures and acts as a sensor of envelope stress, and it has been shown to be important for UPEC virulence (55). Surface structures such as fimbriae are crucial for attachment of UPEC to bladder epithelia (56) as well as abiotic surfaces (57), and UPEC filaments are known to express type 1 fimbriae on their surface, allowing the cells to better adhere to a contact surface in the face of a fluid shear force (22). In this context, it is likely that the Cpx system is activated by the perception of a contact surface by the bacterium (58). Also, in light of studies associating filament formation with decreased antibiotic sensitivity, previous reports of increased antibiotic sensitivity in *cpx* null mutants (55) might point back to a lack of DamX-mediated filamentation in such mutants.

We report here, for the first time, the involvement of DamX in reversible UPEC filamentation, and we demonstrate a clear association between expression of *damX* and morphotype switching in UTI89. Our study suggests an alternative explanation for the filamentous response observed in the late and extracellular stages of the IBC cycle. Our data point to DamX as a key inducer of filamentation at this stage of the life cycle, triggered by the drastic changes under growth conditions occurring in the transition from intracellular proliferation to the urine- and liquid shear-exposed surface growth on the bladder lumen. In addition, neutrophil attack most likely contributes to induction of filamentation via an SOS response, triggered by secreted reactive oxygen species (12).

The molecular mechanism of DamX-mediated cell division inhibition is presently unknown and, given the important clinical implications, further studies aimed at uncovering the DamX morphological switch are warranted.

## MATERIALS AND METHODS

### Ethics statement.

Mice used in this study were handled in accordance with the current guidelines from the National Danish Animal Care Committee. Animal maintenance and experimental protocols were approved by the Ministry of Food, Agriculture and Fisheries, Danish Veterinary and Food Administration (ID 2015-15-0201-00480).

### Bacterial strains and growth conditions.

UTI89, a human cystitis isolate of the O18:K1:H7 serotype (24), was used as the model uropathogenic *E. coli* strain in this study. Deletion strains were constructed in UTI89 by the well-established lambda Red recombinase method devised by Datsenko and Wanner (59) using pKD3 and pKD4 plasmids. Strain UTI89Δ*damX* was constructed using extended regions of complementarity to *damX* 5′ (JMJ553 and JMJ554) and 3′ (JMJ555 and JMJ556) flanking regions on the UTI89 chromosome. Strain UTI89ΔdamX* was constructed using the primers JMJ519 and JMJ991 and confirmed using JMJ491 and JMJ998. All gene deletions were confirmed using gene-specific primers.

Plasmid pSKdamX was derived from a pBAD33 plasmid vector by insertion of a *damX* insert with the *parM* Shine-Dalgarno generated by PCR using primers JMJ777 and JMJ600. The vector and insert were digested using XbaI and HindIII fast digest restriction enzymes (Thermo Scientific) and ligated with T4 DNA ligase (Roche), and the sequence was verified. Plasmid pMAN01 was derived from pSC101 plasmid with a constitutively active promoter driving *gfp* expression. Strains were maintained using 30 µg/ml chloramphenicol (Cml) or 50 µg/ml kanamycin (Kan) as appropriate. All strains and primers are listed in Table S2 in the supplemental material.

UTI89 was cultured at 37°C in Luria-Bertani medium, EZ medium (catalog number m2105; Teknova) without glucose, or in human urine with a USG ranging between 1.025 and 1.030, as suitable. Human urine was filter sterilized, and the USG was measured as detailed previously by Andersen et al. (22). All UTI89 strains were grown in LB medium statically overnight for use in flow chamber and static infection experiments. UTI89 strains used in mouse infections were cultured over two rounds of static growth in LB medium at 37°C as detailed previously (60). Induction and repression of pSKdamX were carried out in EZ medium supplemented with 0.2% arabinose and 0.4% glucose as appropriate.

### Cell culture and infection experiments.

*In vitro* infection experiments were performed on PD07i, an immortalized human bladder epithelial cell line kindly provided by David Klumpp (61). The cell line was maintained in Epilife growth medium (Invitrogen) supplemented with human keratinocyte growth serum (HKGS) and penicillin-streptomycin (Pen/Strep) antibiotics during routine culturing at 5% CO_2_ and 37°C. Epilife medium without Pen/Strep was utilized during infection. Static as well as flow chamber-based infection experiments were carried out as explained in detail previously (22). Briefly, urothelial monolayers cultured for 24 h in tissue culture medium were infected for 20 min by infusion of bacterial cells grown overnight and diluted to an optical density at 600 nm (OD_600_) of 0.2 into the flow chamber at a flow rate of 15 µl/min. Surface colonization and urothelial invasion proceeded in a peptone/glucose medium (antibiotic-free Epilife plus 1% HKGS with 0.5% peptone and 0.5% glucose) for ~9 h before removal of extracellular bacteria by addition of 100 µg/ml gentamicin to the medium for 20 h. Bacterial filamentation and secondary surface colonization were induced by shifting from the growth medium to concentrated urine (USG, >1.025) for 22 to 24 h. Secondary surface-colonizing bacteria were eluted by detaching the pump and elevating the medium flask to increase hydrostatic pressure and manually tapping the inlet tubing to apply adequate shearing force to release bacteria from the cell layers.

### Mouse strain and infection.

Eight-week-old female C3H/HeN mice (Janvier Labs, France) were inoculated with 50 µl of a culture with an OD_600_ of 1 (~5 × 10^8^ bacteria) by transurethral catheterization. Bladders were harvested 18 h postinfection to be bisected, splayed on silicone pads, and fixed in 3% paraformaldehyde solution for microscopy. Urine from infected mice was collected immediately prior to tissue harvest and fixed using 10% formalin buffer. Bladders for CFU counts were harvested 18 h, 48 h, and 14 days postinfeciton, homogenized, and suitably diluted (10, 10^−2^, 10^−3^, and 10^−4^) for CFU plating. Each UTI89 strain was tested on a group of 5 mice, and the experiments were performed twice in their entirety. Mouse infection and sample preparation were performed in strict adherence to previously published protocols reported by Hung et al. (60).

### DamX-induced filament formation.

Induction of filamentation in strain UTI89/pSKdamX was performed by resuspending bacteria grown overnight on LB agar plates with 0.4% glucose and 30 µg/ml Cml in sterile phosphate-buffered saline (PBS) to an OD_600_ of 0.1 and seeding on sterile glass slides within flow chambers for 20 min at a flow rate of 100 µl/min. Flow chambers were subjected to a flow of EZ medium with 0.2% arabinose and 30 µg/ml Cml at 37°C at a constant flow rate of 50 µl/min and observed for filamentation. Reintroduction of 0.4% glucose to the EZ medium was used to force reversal of filaments in the flow chambers. Strains MG1655/pSKdamX and UTI89/pSKdamX were cultured on both LA plates and LB broth in the presence of the inducer (0.2% arabinose) or repressor (0.4% glucose) as appropriate, to ascertain phenotypic differences.

### Microarray sample preparation.

UTI89 was grown overnight at 37°C in both LB medium and urine as reference conditions for analysis. Bacteria were pelleted by centrifugation, washed with sterile PBS, and frozen in liquid nitrogen. UTI89 was harvested from the flow chambers at the intracellular phase by carefully dismantling the chambers to obtain the glass slides with infected bladder cells. Lysing of the infected PD07i cells was performed by pipetting 0.1% Triton X-100 (reconstituted in sterile PBS) directly onto the glass slides that had been placed in a sterile petri dish, and the lysate was collected in sterile Falcon tubes. Intracellular bacteria were washed with sterile PBS, pelleted, and frozen in liquid nitrogen. Filaments formed within the flow chambers were flushed out of the chambers and frozen in liquid nitrogen after one wash with sterile PBS. For the filament reversal phase, harvested filaments were maintained at 37°C in a 1:1 suspension of antibiotic-free HKGS-supplemented Epilife medium and PBS for 3 h in accordance with earlier studies (22). The reverted rod-shaped bacteria were examined microscopically to ensure reversal, pelleted by centrifugation, and frozen. Biological triplicates were used for each phase of UTI89 infection. RNA isolation was performed using heavy phase-lock gel tubes (5Prime), and total RNA was dissolved in RNase-free water. RiboGuard (Epicenter) was used to inhibit RNA degradation. Subsequent cDNA synthesis and Cy3 labeling were performed using the Low Input Quick Amp labeling kit (Agilent Technologies) as per the manufacturer’s recommendations.

### Microarray design and analysis.

Custom-designed 8-by-60,000 SurePrint G3 arrays from Agilent Technologies were used for microarray analysis. The arrays were designed to cover at least two independent probes per gene as well as the intergenic regions of the UTI89 genome in four replicates and 1,319 control probes for detection of hybridization efficiency as well as eukaryotic control probes, using the eArray system (Agilent Technologies). Hybridization was carried out using a gene expression hybridization kit and wash buffers (Agilent Technologies) as per the manufacturer’s recommendations and scanned using an Agilent scanner. Feature-extracted files were analyzed using DNAStar software. Probe signal intensities were subjected to background correction, quantile normalization, and median polish. To identify differentially expressed transcripts, the log_2_ fold change was calculated (cutoff, ≥2 log_2_ fold change), and filtering was based on Student’s *t* test (significance cutoff, *P* < 0.05). The statistically significant, differentially expressed transcripts were further examined based on gene ontology information obtained from EcoCyc and literature searches.

### Real-time PCR.

Primers for real-time PCR, listed in Table S2c in the supplemental material, were designed using Primer3 software. Total RNA was isolated from each infection phase following the same protocol as for microarray sample preparation. Total RNA from UTI89 (wild type) and strain UTI89Δ*damX* (see Fig. S3a in the supplemental material) was harvested from exponentially growing bacteria in culture. One microgram of total RNA was DNase treated for 30 min at 37°C and heat inactivated, then used for cDNA synthesis using a Maxima first-strand cDNA synthesis kit (Thermo Scientific). Real-time quantification was performed using SYBR Select master mix (Life Technologies) as per the recommended protocol. Levels of *rrsA* were used as an internal control, and real-time PCRs were run in 96-well plates in an Mx3005P QPCR system (Agilent Technologies).

### Flow cytometry.

Flow cytometry was performed on a BD FACSAria II flow cytometer (Becton, Dickinson and Company). UTI89 cells grown to exponential phase as normal rod-shaped bacteria and filamentous UTI89 obtained from an *in vitro* flow chamber infection were fixed in 3.7% paraformaldehyde prior to flow cytometry. Data from 10^5^ events per sample were collected and analyzed using BD FACSDiva software (Becton, Dickinson and Company). UTI89 from the two conditions was distinguished on the basis of FSC-A and SSC-A on the *x* and *y* axes, respectively. Gating was performed using normal rod-shaped bacteria, such that all bacterial events occurring outside gate P1 were designated “not P1” and corresponded to cells longer than normal rod-shaped bacteria.

### Microscopy.

Each individual phase of UTI89 infection was confirmed by microscopic inspection. All phase-contrast microscopy using UTI89 deletion strains during flow chamber infection was performed using a Leica DMRE fluorescence microscope. Time-lapse imaging of filament induction as well as mouse bladder and urine imaging were performed with the Olympus cellSens Dimension platform v. 1.7 where, using a 40× objective, composite images obtained by collapsing 26 *Z*-stack slices with a step size of 0.5 µm were captured at 10-min intervals. Contrast adjustment and scale bars were added using ImageJ software.

### Western blotting.

UTI89 filaments and strain UTI89/pSKdamX samples were obtained by flushing bacteria out of the flow chamber system and snap-freezing. The bacterial cell pellets were resuspended in 1× SDS loading buffer (60 mm Tris-HCl [pH 6.8], 2% SDS, 10% glycerol, 0.005% bromophenol blue, 5 mm EDTA, 0.1 M dithiothreitol) and boiled for 5 min at 90°C with vigorous agitation. Protein from ~10^8^ cells was loaded and separated on 4-to-12% Invitrogen NuPage Novex bis-Tris minigels as detailed by Boysen et al. (62). The proteins were transferred onto polyvinylidene difluoride membranes (Amersham Hybond) that were probed with a 1:1,000 dilution of anti-FtsZ (AgriSera), 1:1,000 dilution of anti-DamX (35), or 1:10,000 anti-GroEL antibodies, followed by appropriate secondary antibodies. Band quantification was performed using Image Studio Lite v4 software.

### Plasmid stability assay.

Strains UTI89/pKD46 and UTI89Δ*damX/*pKD46 were grown overnight with aeration at 30°C in LB plus 100 µg/ml Amp and used to subculture a starter culture in fresh LB plus 100 µg/ml Amp at 30°C to an OD_600_ of 0.3. The starter culture was diluted to an OD_600_ of 0.03 in LB at 37°C and sampled every 30 min for CFU plating on LB agar (LA) and LA plus 100 µg/ml Amp plates. Cultures at 30°C in LB plus 100 µg/ml Amp were also used in a flow chamber-based infection of PD07i bladder epithelial cells. Flow chamber-based infection was carried out as before, without any Amp selection. A 100-µg/ml concentration of gentamicin was added 9 hpi, and the infection experiment was terminated after 24 hpi. The chambers were subjected to three short 5-min bursts at a higher flow rate (50 µl/min) to wash away extracellular bacteria and subsequently dismantled to obtain the glass slide with infected bladder cells. The infected epithelial cells were trypsinized and lysed with 0.1% Triton. Intracellular bacteria from infected bladder cells were harvested for CFU plating on LA and LA plus 100 µg/ml Amp plates. CFU counts from LA and LA plus 100 µg/ml Amp plates were used to calculate percentages of plasmid retention over time {100 × [(CFU with LA plus Amp)/(CFU with LA)]}.

### Statistical analysis.

All infection and real-time reverse transcription-PCR experiments were conducted on biological triplicates, and mean values were plotted along with standard deviations. Student’s *t* test was used to calculate *P* values and determine statistical significance in stationary infections by using GraphPad Prism 5.01 software. A Mann-Whitney unpaired test was used to calculate the significance of mouse CFU counts using GraphPad Prism 5.01 software. *P* values less than 0.05 were defined significant for all experiments.

### Microarray data accession number.

Array data have been deposited in ArrayExpress (accession number E-MTAB-3611).

## SUPPLEMENTAL MATERIAL

Video S1a  Time-lapse microscopy illustrating the effect of *damX* overexpression. The video shows time-lapse live-cell imaging, using a 40× objective. Strain UTI89/pSKdamX was seeded on glass slides in flow chambers and imaged at 10-min intervals using 0.2% arabinose from the 0-min time point, followed by subsequent addition of 0.4% glucose at the 270-min time point. Download Video S1a, AVI file, 1 MB

Video S1b  Time-lapse microscopy of the uninduced negative control. The video shows time-lapse live-cell imaging using a 40× objective. Strain UTI89/pSKdamX was continually exposed to 0.4% glucose for the entire duration of imaging to serve as a negative control for *damX* overexpression. Download Video S1b, AVI file, 0.4 MB

Video S1c  Time-lapse microscopy of the induced vector control. The video shows time-lapse live-cell imaging using a 40× objective. Strain UTI89/pBAD33 was continually exposed to 0.2% arabinose for the entire duration of imaging to serve as a vector control. Download Video S1c, AVI file, 0.4 MB

Figure S1  Study design and temporal expression of cell division genes (a) Strain UTI89wt cultured in LB medium was used to infect bladder epithelial cells grown on glass slides as part of the flow chamber infection model. LB medium and concentrated urine cultures served as reference conditions. UTI89 was subsequently harvested as intracellular bacteria within infected epithelial cells, filaments fluxed from infected bladder epithelial cells post-urine exposure, and filaments that reverted to normal rod-shaped bacteria. RNA from each phase was processed and used as input in transcriptomic analysis using microarray technology. (b) Heat map of the signal intensities of genes associated with cell division across all infection phases relative to the reference condition of UTI89 cultured in urine. The color gradient depicted in the legend represents the most downregulated genes (blue, ≤2 log_2_ fold change) to the most upregulated (red, ≥2 log_2_ fold change). (c) Graphical representation of the gene ontological distribution of the statistically significant genes (*P ≤* 0.05) that were differentially expressed (log_2_ fold change, ≥2) between intracellular, filamentous, and reversal phases compared to the urine-grown culture reference. Download Figure S1, PDF file, 2.1 MB

Figure S2  Growth rate measurements. (a) Strains UTI89wt, UTI89Δ*damX*, UTI89Δ*cedA*, UTI89Δ*slmA*, and UTI89Δ*zapB* were grown in LB broth (black) or concentrated urine (red) in order to compare growth rates. Mean optical density measurements (at 600 nm) were recorded for the cultures harvested once every hour. Mean values from biological triplicates of each strain and their standard deviations were plotted to construct a growth curve on the log_10_ scale. The panel below lists doubling times of the strains during exponential growth in LB medium. (b and c) Stationary infection was carried out in the human urinary bladder-derived PD07i cell line with strains UTI89wt and UTI89Δ*damX*. No significant difference was observed between the strains in their ability to adhere (b) or invade (c) bladder epithelial cells. (d) The percent retention of pKD46 plasmid in strains UTI89 and UTI89Δ*damX* harvested from infected bladder cells in flow chambers (gray) was found to be 70% and 72%, respectively. The initial infection input (white) grown at 30°C with ampicillin is shown as white bars. (e) The percentage retention (red) of pKD46 measured along the right *y* axis and growth curve (black) along the left *y* axis were plotted over time to create a standard curve of plasmid retention. The dotted lines intersecting the right *y* and *x* axes represent the intracellular percent retention of 70% in strain UTI89 and 72% in strain UTI89Δ*damX* relative to the standard percent retention curve. Download Figure S2, PDF file, 1 MB

Figure S3  Investigation of possible polarity effects of *damX* deletion. (a) The relative expression levels of *dam* operon genes were examined by real-time PCR. The graph presents mean expression values for *damX*, *aroB*, *aroK*, and *dam* with standard deviations for strain UTI89Δ*damX* relative to the UTI89 wild type from biological triplicates. The dotted line represents expression levels that were set to 1.0 for strain UTI89wt. (b) Flow chamber-based infection of the PD07i bladder cell line was carried out with strain UTI89ΔdamX*. Panels in the top row represent strain UTI89wt biofilm formation (cloudy appearance) on exposure to concentrated urine and strain UTI89wt filaments. The bottom row depicts strain UTI89ΔdamX* surface biofilm and an absence of filamentation. Scale bars, 20 µm. Download Figure S3, PDF file, 2.3 MB

Figure S4  Strain UTI89Δ*sulA* filaments observed in urine of infected mice 18 hpi. (a) Splayed urinary bladders of C3H/HeN mice infected with strain UTI89Δ*sulA*/pMAN01 were examined for IBCs and filaments 18 hpi. (b and c) Urine collected from infected bladders prior to tissue harvest was also examined and revealed bacterial filaments. Scale bars, 10 µm. Download Figure S4, PDF file, 0.9 MB

Figure S5  Comparison of *damX* overexpression in K-12 and UPEC. Strains MG1655/pSKdamX (K-12) and UTI89/pSKdamX (UPEC) were cultured on LA plates where *damX* expression was repressed with 0.4% glucose (top row), on LA plates with 0.2% arabinose where *damX* was overexpressed (middle row), and in LB broth with 0.2% arabinose (bottom row). The two LA plates depicted at the bottom were supplemented with 0.4% glucose (left) and 0.2% arabinose (right). Both plates were divided in half and strain MG1655/pSKdamX was fine streaked on the left and strain UTI89/pSKdamX is shown on the right half. Download Figure S5, PDF file, 2.8 MB

Table S1  Significant genes from different phases of UTI89 infection. Table S1, XLSX file, 0.1 MB

Table S2  Strains, primers, and reverse transcription-PCR primers used in the study. Table S2, PDF file, 0.1 MB
