# Supplementary figures and images for: DamX Controls Reversible Cell Morphology Switching in Uropathogenic Escherichia coli
Source: mBio. 2016 Aug 2;7(4):e00642-16. doi: 10.1128/mBio.00642-16 (PMC4981707; doi:10.1128/mBio.00642-16)

Figure S1

a.

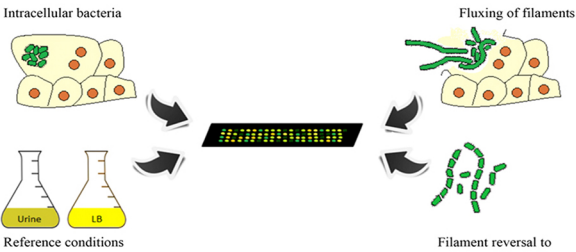

b.

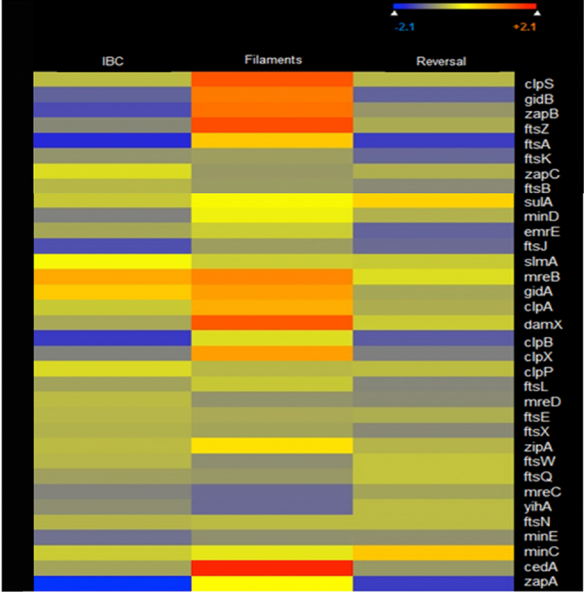

c.

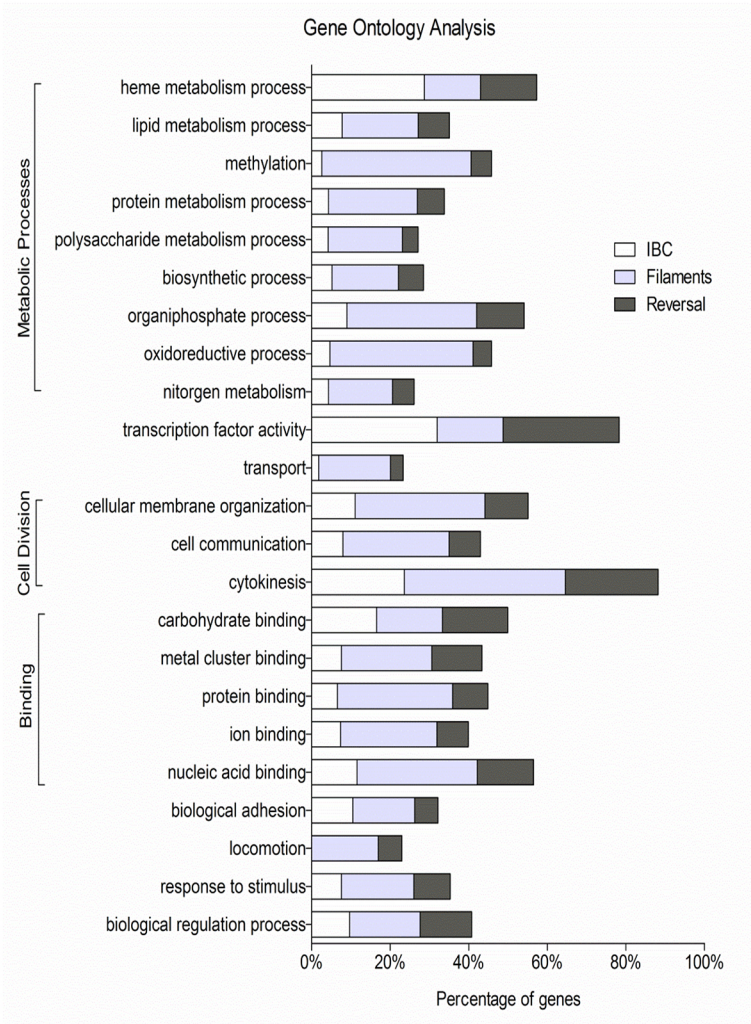

Supplement: Figure S1 — Study design and temporal expression of cell division genes (a) Strain UTI89wt cultured in LB medium was used to infect bladder epithelial cells grown on glass slides as part of the flow chamber infection model. LB medium and concentrated urine cultures served as reference conditions. UTI89 was subsequently harvested as intracellular bacteria within infected epithelial cells, filaments fluxed from infected bladder epithelial cells post-urine exposure, and filaments that reverted to normal rod-shaped bacteria. RNA from each phase was processed and used as input in transcriptomic analysis using microarray technology. (b) Heat map of the signal intensities of genes associated with cell division across all infection phases relative to the reference condition of UTI89 cultured in urine. The color gradient depicted in the legend represents the most downregulated genes (blue, ≤2 log2 fold change) to the most upregulated (red, ≥2 log2 fold change). (c) Graphical representation of the gene ontological distribution of the statistically significant genes (P ≤ 0.05) that were differentially expressed (log2 fold change, ≥2) between intracellular, filamentous, and reversal phases compared to the urine-grown culture reference. Download [file mbo004162928sf1.pdf]

Figure S2

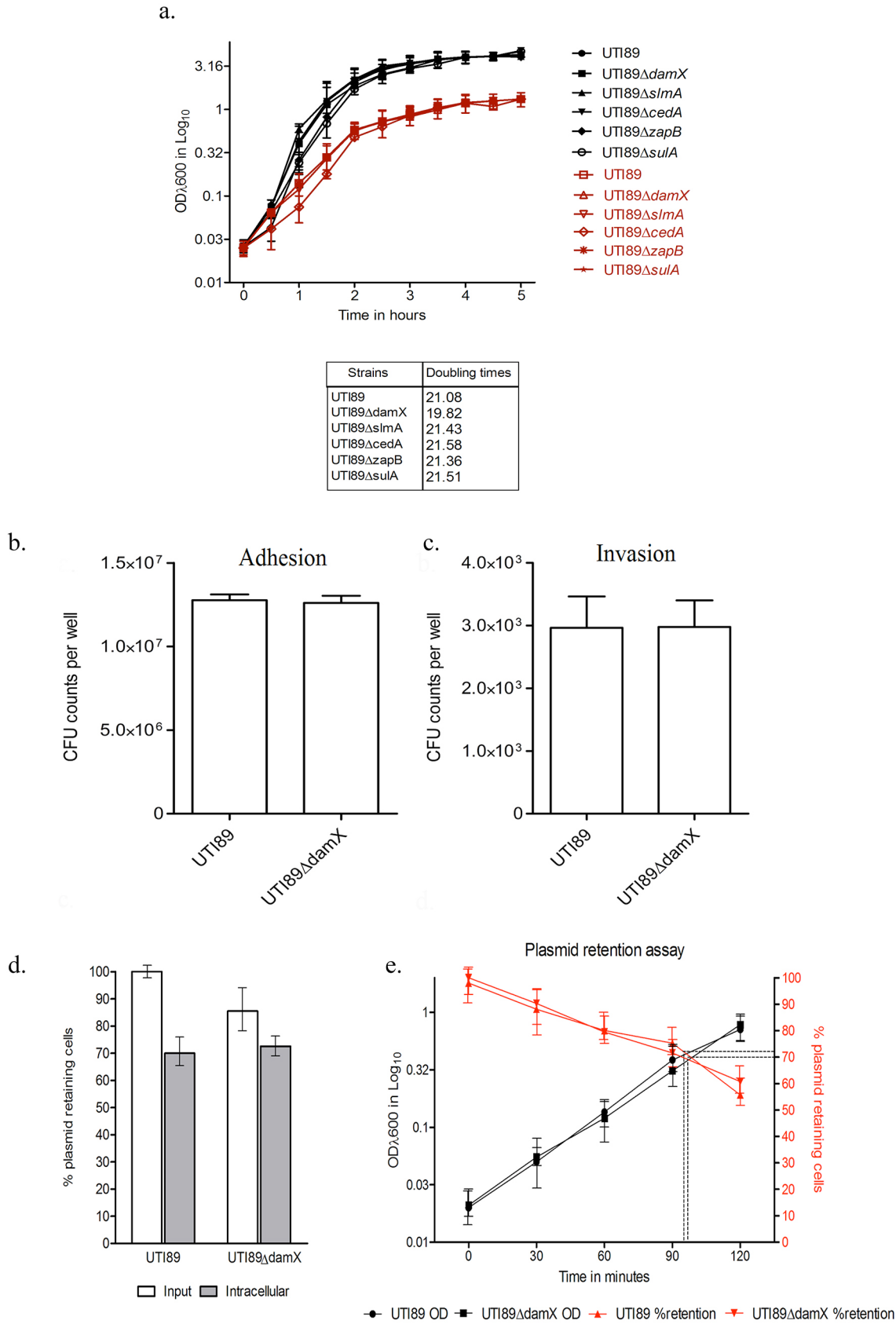

Supplement: Figure S2 — Growth rate measurements. (a) Strains UTI89wt, UTI89ΔdamX, UTI89ΔcedA, UTI89ΔslmA, and UTI89ΔzapB were grown in LB broth (black) or concentrated urine (red) in order to compare growth rates. Mean optical density measurements (at 600 nm) were recorded for the cultures harvested once every hour. Mean values from biological triplicates of each strain and their standard deviations were plotted to construct a growth curve on the log10 scale. The panel below lists doubling times of the strains during exponential growth in LB medium. (b and c) Stationary infection was carried out in the human urinary bladder-derived PD07i cell line with strains UTI89wt and UTI89ΔdamX. No significant difference was observed between the strains in their ability to adhere (b) or invade (c) bladder epithelial cells. (d) The percent retention of pKD46 plasmid in strains UTI89 and UTI89ΔdamX harvested from infected bladder cells in flow chambers (gray) was found to be 70% and 72%, respectively. The initial infection input (white) grown at 30°C with ampicillin is shown as white bars. (e) The percentage retention (red) of pKD46 measured along the right y axis and growth curve (black) along the left y axis were plotted over time to create a standard curve of plasmid retention. The dotted lines intersecting the right y and x axes represent the intracellular percent retention of 70% in strain UTI89 and 72% in strain UTI89ΔdamX relative to the standard percent retention curve. Download [file mbo004162928sf2.pdf]

a.

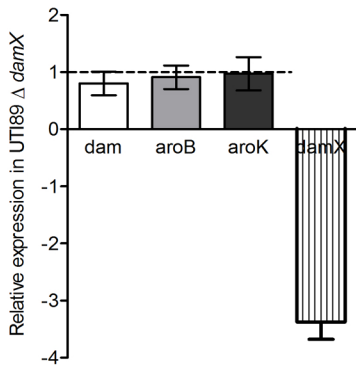

b.

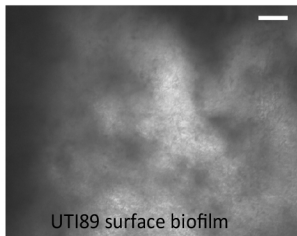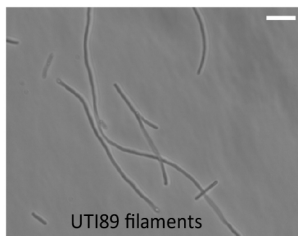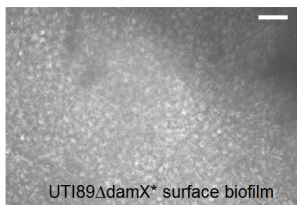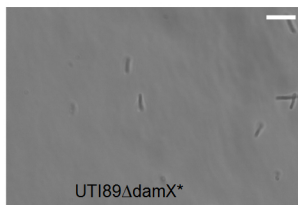

Supplement: Figure S3 — Investigation of possible polarity effects of damX deletion. (a) The relative expression levels of dam operon genes were examined by real-time PCR. The graph presents mean expression values for damX, aroB, aroK, and dam with standard deviations for strain UTI89ΔdamX relative to the UTI89 wild type from biological triplicates. The dotted line represents expression levels that were set to 1.0 for strain UTI89wt. (b) Flow chamber-based infection of the PD07i bladder cell line was carried out with strain UTI89ΔdamX*. Panels in the top row represent strain UTI89wt biofilm formation (cloudy appearance) on exposure to concentrated urine and strain UTI89wt filaments. The bottom row depicts strain UTI89ΔdamX* surface biofilm and an absence of filamentation. Scale bars, 20 µm. Download [file mbo004162928sf3.pdf]

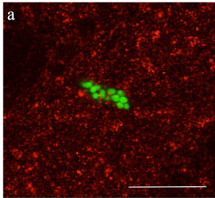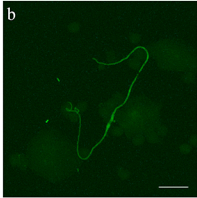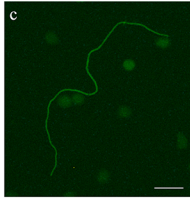

Supplement: Figure S4 — Strain UTI89ΔsulA filaments observed in urine of infected mice 18 hpi. (a) Splayed urinary bladders of C3H/HeN mice infected with strain UTI89ΔsulA/pMAN01 were examined for IBCs and filaments 18 hpi. (b and c) Urine collected from infected bladders prior to tissue harvest was also examined and revealed bacterial filaments. Scale bars, 10 µm. Download [file mbo004162928sf4.pdf]

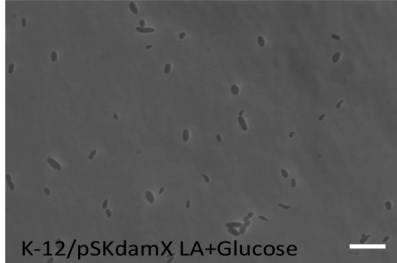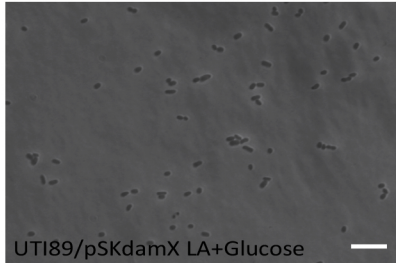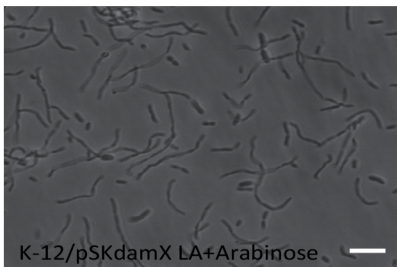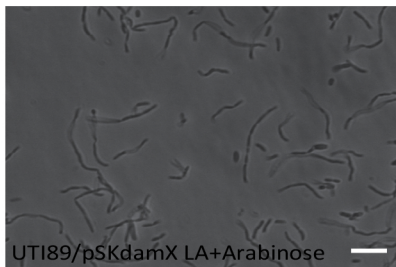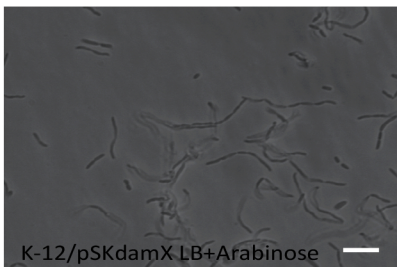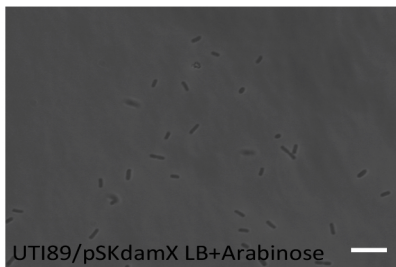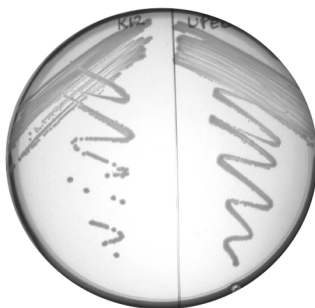

LB-Agar+Glucose

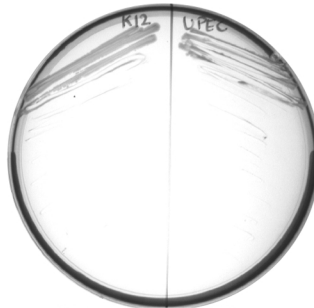

LB-Agar+Arabinose

Supplement: Figure S5 — Comparison of damX overexpression in K-12 and UPEC. Strains MG1655/pSKdamX (K-12) and UTI89/pSKdamX (UPEC) were cultured on LA plates where damX expression was repressed with 0.4% glucose (top row), on LA plates with 0.2% arabinose where damX was overexpressed (middle row), and in LB broth with 0.2% arabinose (bottom row). The two LA plates depicted at the bottom were supplemented with 0.4% glucose (left) and 0.2% arabinose (right). Both plates were divided in half and strain MG1655/pSKdamX was fine streaked on the left and strain UTI89/pSKdamX is shown on the right half. Download [file mbo004162928sf5.pdf]
