# Supplementary material for: DamX Controls Reversible Cell Morphology Switching in Uropathogenic Escherichia coli
Source: mBio. 2016 Aug 2;7(4):e00642-16. doi: 10.1128/mBio.00642-16 (PMC4981707; doi:10.1128/mBio.00642-16)
Supplement: Table S2 — Strains, primers, and reverse transcription-PCR primers used in the study. [file mbo004162928st2.pdf]

Supplementary Table 2a

Strains	Genotype	Source
UTI89	Cystitis-derived isolate, serotype O18:K1:H7	[1]
UTI89Δ*damX*	Δ*damX*, Cml^R	This study
UTI89Δ*damX*^{short}	Δ*damX*, Kan^R	This study
UTI89Δ*slmA*	Δ*slmA*, Kan^R	This study
UTI89Δ*zapB*	Δ*zapB*, Cml^R	This study
UTI89Δ*cedA*	Δ*cedA*, Cml^R	This study
UTI89Δ*sulA*	Δ*sulA*, Cml^R	[2]
UTI89/pSKdamx	pBAD33; pBAD::*damX*^{parM}	This study
MG1655/pSKdamX	pBAD33; pBAD::*damX*^{parM}	This study
UTI89/pMAN01	UTI89, P_{LtetO} ::*gfp*	This study
UTI89Δ*damX*/pMAN01	Δ*damX*, P_{LtetO} ::*gfp*	This study
UTI89Δ*sulA*/pMAN01	Δ*sulA*, P_{LtetO} ::*gfp*	This study
UTI89/pOU82	*oriR1*, Amp^R	This study
UTI89Δ*damX* /pOU82	Δ*damX, oriR1*, Amp^R	This study
**Plasmids**	**Genotype**	
pKD3	Cml^R, template plasmid	[3]
pKD4	Kan^R, template plasmid	
pKD46	Bla, λ Red recombinase expression plasmid	
pBAD33	p15; *araC* pBAD, Cml^R	[4]
pMAN01	pSC101 derivative, constitutive P_{LtetO} ::*gfp*, Cml^R	
pOU82	*oriR1*, Amp^R	[5]

1. Mulvey, M.A., J.D. Schilling, and S.J. Hultgren, *Establishment of a persistent Escherichia coli reservoir during the acute phase of a bladder infection.* Infect Immun, 2001. **69**(7): p. 4572-9.
2. Andersen, T.E., et al., *Escherichia coli uropathogenesis in vitro: invasion, cellular escape, and secondary infection analyzed in a human bladder cell infection model.* Infect Immun, 2012. **80**(5): p. 1858-67.
3. Datsenko, K.A. and B.L. Wanner, *One-step inactivation of chromosomal genes in Escherichia coli K-12 using PCR products.* Proc Natl Acad Sci U S A, 2000. **97**(12): p. 6640-5.
4. Guzman, L.M., et al., *Tight regulation, modulation, and high-level expression by vectors containing the arabinose PBAD promoter.* J Bacteriol, 1995. **177**(14): p. 4121-30.

5. Jensen, R.B., et al., *Comparison of ccd of F, parDE of RP4, and parD of R1 using a novel conditional replication control system of plasmid R1.* Mol Microbiol, 1995. **17**(2): p. 211-20.

Supplementary Table 2b

Primers	Description	Sequence
JMJ519	Δ*damX*_KO_F	TCAAACCAGAAGACGAGCTGAAACCCGATCCCAGCGATCGTCGTACTGGTCGTTCTCGTCAATCTTCTGAGTGTAGGCTGGAGCTGCTTC
JMJ520	Δ*damX*_KO_R	TTTTGGCCTGGACATCTGCTGGCAATGTAGATACCGCTTTTTTCGCCTCTTCTTTCGAAGCATACACGCCCATATGAATATCCTCCTTA
JMJ991	Δ*damX*_KO_R2	ATTGACCGCCACATTGTTCAACTGTTGCTGATTTTGTGGCTGGGTCAGCGCATTGTTCAGGTCACCCTGCCATATGAATATCCTCCTTA
JMJ553	Δ*damX*_5'_F	ACCTTTGGTCATGCCATTGAAG
JMJ554	Δ*damX*_5'_R	ACGACCAGTACGACGATCGCTGG
JMJ555	Δ*damX*_3'_F	GTTTACTATCAGTTCCGCGAAGAGTTC
JMJ556	Δ*damX*_3'_R	CTGGACATCTGCTGGCAATGTAGA
JMJ491	Δ*damX*_F	AGAAAGGTCAGGCCGCTTAT
JMJ492	Δ*damX*_R	GAGAAAGCTCCGACAGCATC
JMJ998	Δ*damX*_R2	GGGTTCAGTCGGCAATGTAG
JMJ314	Δ*slmA*_KO_F	GCGGTCATAGCGTGGGTGCCGCCTGGCAAGTGCTTATTTTCAGGGGTATTTTGTAACATGGTGTAGGCTGGAGCTGCTTCG
JMJ 315	Δ*slmA*_KO_R	TAAAGAAACTCGCCGGATGAAAAGTCATCCGGCGTCATATTACTGCAACTGTGCAGCAATCATATGAATATCCTCCTTAGTTCC
JMJ316	Δ*slmA*_F	CGCATCCGAATAACGTCATAAC
JMJ317	Δ*slmA*_R	GGTTAAGGCCTATCGCGAAGAG
JMJ497	Δ*zapB*_KO_F	GACAATGTCATTAGAAGTGTTTGAGAAACTGGAAGCAAAAGTACAGCAGGCGATTGATACCATCACTCTGGTGTAGGCTGGAGCTGCTTC
JMJ498	Δ*zapB*_KO_R	TGCGACCCAGCAGGGCCTGTAGACGTTCCTGCCAGCCGTTCTGCTGTTCTTTCAGATGGTTGTTCTCACGCATATGAATATCCTCCTTA
JMJ499	Δ*zapB*_F	GGTAATCGGGACGAGGATTT
JMJ500	Δ*zapB*_R	TTTTTCTGCGTTACCTGTTGG
JMJ493	Δ*cedA*_KO_F	TGAAGCCAGTAATGAAGAAACCGCTCCGTCAGCAAAACCGCCAGATTATTAGCTATGTCCCACGCACGGAGTGTAGGCTGGAGCTGCTTC
JMJ494	Δ*cedA*_KO_R	CATTTTCCTGACGAATCTGATTTGCCCAACGCTGGGCGGATTCAGGCACAGTAAACGCCGGTGAGCGCAGCATATGAATATCCTCCTTA
JMJ495	Δ*cedA*_F	CAGGACGTACAGGGGCTAAA
JMJ496	Δ*cedA*_R	TGGCACACCGTTAATTCTGA
JMJ777	pSKdamX_F	CCCCCCTCTAGACACCATAAGGAGTTTTATAAATGGATGAATTCAAACCAGAAGACG
JMJ600	pSKdamX_R	CCCTCGGAAGCTTGCGGGAGATAACCTTGATTA
JMJ167	pBAD33_F	ATCACGGCAGAAAAGTCCAC
JMJ168	pBAD33_R	GCCAGGCAAATTCTGTTTTATC

Supplementary Table 2c

RT-PCR primers	Sequence
*rrsA*_F	GTCGAACGGTAACAGGAAGAAG
*rrsA*_R	GGCAGTTTCCCAGACATTACTC
*ftsZ*_F	GGTATCCTGACCGTTGCTGT
*ftsZ*_R	TTTCAGCAGTTTGTCGTTCG
*ftsA*_F	GGCAGAATTGATGGCAGATT
*ftsA*_R	GGACGACGTTTTCCACATCT
*zipA*_F	ACTTCAAGCTGATGCTGCAA
*zipA*_R	GCGGATGATGTCCTGGTACT
*zapA*_F	AAAGGGATGCGTTGAATCAG
*zapA*_R	CGCTTTTTCTTGCGCTAACT
*zapB*_F	GCAGATGGAAATCGAAGAGC
*zapB*_R	AGCCGTTCTGCTGTTCTTTC
*damX*_F	ACCACGTCTGCGGAAAATAC
*damX*_R	AGGTCACCCTGCACTTCAAC
*cedA*_F	CGTTTCGTGATGTCTGGATG
*cedA*_R	ACGAATCTGATTTGCCCAAC
*sulA*_F	TCTGGGCTACCCTTAACGAA
*sulA*_R	TCAAATCATCTGCCAACCAA
*gidB*_F	TCGCTGAAGGTCTGGTTGAT
*gidB*_R	CACTCACGCGTTAACATCGT
*aroB*_F	GAT ACTACTCTGGTGGCGCT
*aroB*_R	GTACACCGCGCTGATAACTC
*aroK*_F	GTTGCTGCACGTTGAAACAC
*aroK*_R	GTCACGTCGGCAATCTCTTC
*dam*_F	TCGCTGAAGGTCTGGTTGAT
*dam*_R	CACTCACGCGTTAACATCGT
